# Blood pressure parameters and the risk of chronic limb-threatening ischemia: The Singapore Chinese Health Study

**DOI:** 10.1016/j.jvs.2025.12.088

**Published:** 2025-12-12

**Authors:** Ariel Fangting Ying, Mohammad Talaei, Shan Zhang, Woon-Puay Koh

**Affiliations:** a Cardiovascular and Metabolic Disorders Program, Duke-National University of Singapore Medical School, Singapore;; b Centre for Preventive Neurology, Wolfson Institute of Population Health, Queen Mary University of London, London;; c Department of Health Policy and Management, School of Public Health, Peking University, Beijing;; d Healthy Longevity Translational Research Programme, Yong Loo Lin School of Medicine, National University of Singapore, Singapore;; e ASTAR Institute for Human Development and Potential, Singapore.

**Keywords:** Peripheral artery disease, Vascular epidemiology, Hypertension, Chronic limb-threatening ischemia, Blood pressure, Pulse pressure

## Abstract

**Background:**

Although hypertension is an established risk factor for chronic limb-threatening ischemia (CLTI), little is known about how the individual blood pressure (BP) parameters could influence CLTI risk. We investigated associations of systolic BP, diastolic BP, and pulse pressure (PP) with CLTI risk in a prospective population-based cohort.

**Methods:**

We used data from the Singapore Chinese Health Study, involving 30,636 participants aged 46–85 years, with BP measured by trained interviewers during follow-up I interviews from 1999 to 2004. CLTI cases were identified via linkage to hospital records. Associations between various BP parameters and CLTI risk were examined via multivariable Cox models.

**Results:**

After a mean follow-up of 13.6 years, there were 407 incident CLTI cases. In individual models, all BP parameters were positively associated with CLTI risk in a stepwise manner. However, after pairwise adjustment for PP, systolic and diastolic BP were no longer associated with CLTI risk. Positive associations between different grades of hypertension and CLTI risk were also attenuated after adjusting for PP. Conversely, after adjustment for other BP parameters and vascular risk factors, PP remained independently associated with CLTI risk in a stepwise manner (*P* for trend ≤ .002). Compared with the lowest PP category (<40 mm Hg), the hazard ratio (95% confidence interval) was 5.24 (3.04–9.03) for the highest category (≥80 mm Hg).

**Conclusions:**

Our study found that only PP remained independently associated with CLTI risk after adjusting for other BP parameters and provided a basis for targeting reduction of arterial stiffness to decrease CLTI risk. (J Vasc Surg 2026;83:1183–91.)

Chronic limb-threatening ischemia (CLTI), the most severe form of peripheral artery disease (PAD), is a progressive atherosclerotic disease characterized by the presence of nonhealing ulcers for more than two weeks, ischemic rest pain, and/or gangrene.^1,2^ Clinical management of CLTI frequently requires urgent vascular reconstruction either via surgical or endovascular techniques to restore blood flow. If revascularization fails, patients may require lower extremity amputation (LEA), resulting in a high morbidity burden and a high risk of mortality.^3,4^ In fact, the 5-year mortality rate for CLTI has been reported to be approximately 50%.^5^

Studies have shown that PAD worldwide has risen by around 25% over a period of 10 years from 2000 to 2010 and that this steep rise is expected to persist due to population aging and increasing prevalence of vascular risk factors, with hypertension being one of the most important factors.^6^ In fact, in many cohorts studying CLTI, a prevalence of at least 40% for hypertension has been reported among the cases.^7–9^ As such, evaluating blood pressure (BP) parameters is of critical importance in understanding the development of CLTI.

BP parameters commonly used in clinical practice include systolic BP (SBP) and diastolic BP (DBP), as well as pulse pressure (PP) derived as the difference between SBP and DBP. Although both SBP and DBP are important for assessing cardiovascular risk, it has been observed that SBP and DBP will increase with age almost in parallel until around the age of 55 years, after which SBP will continue to rise, whereas DBP will begin to fall.^10,11^ Hence, an increase in PP could be a better marker of age-related arterial wall degeneration and arterial wall stiffness than either SBP or DBP alone.^12^ Indeed, recent research has increasingly noted that a widened PP is significantly associated with the risk of cardiovascular disease independently of the traditional indices of SBP and DBP,^13^ which suggested that it is a better predictor of coronary heart disease risk than SBP or DBP in older adults.^14^

In contrast, although some cohort studies have examined the relationship between PP and PAD risk, less is known about how the individual BP parameters could influence CLTI risk. A population-based prospective study in China reported that a higher PP was associated with an increased risk of incident PAD as defined by an ankle-brachial index value of <0.9, after adjusting for hypertension and other cardiovascular risk factors.^15^ However, the authors did not examine whether this association was confounded or mediated by elevated SBP or reduced DBP. Similar results were reported by another study that used data from the Chronic Renal Insufficiency Cohort, but again, the authors only adjusted for hypertension and other traditional cardiovascular risk factors and did not account for the relationship between PP and SBP or DBP.^16^

To the best of our knowledge, only two studies on PP and PAD have included the effects of SBP and DBP, but with conflicting results. One showed that SBP was slightly superior to PP in predicting the risk of PAD,^14^ whereas another study showed that PP was predictive for ankle-brachial index independent of SBP or DBP.^17^ However, both studies were restricted to patients with diabetes, used a wide range of possible outcomes including asymptomatic PAD diagnosed via ankle-brachial index, and were either cross-sectional in design or had a short follow-up period, thus rending it difficult to draw conclusions on temporal relationships between PP and the risk of severe PAD necessitating treatment in the general populace.

Therefore, in the present study, we investigated the association of SBP, DBP, and PP with CLTI risk in a large, prospective, population-based cohort of Chinese adults residing in Singapore. We further assessed how different grades of hypertension, as defined by the 2017 American College of Cardiology/American Heart Association (ACC/AHA) criteria,^17^ were associated with CLTI risk, and whether any such association could be explained by an elevated PP.

## METHODS

### Study population.

The Singapore Chinese Health Study was a large prospective population-based cohort study established in 1993–1998 via the recruitment of 63,257 Chinese adults (27,959 men and 35,298 women) aged 45–74 years at baseline and described in detail elsewhere.^18^ Study participants were permanent residents or citizens of Singapore who resided in government purpose-built housing estates, which comprised 86% of the population at that time. Follow-up I interviews were conducted via telephone from 1999 to 2004. Among the 52,322 participants contacted successfully during this follow-up period, trained research personnel visited 30,636 consenting participants in their homes to measure their BP and to collect blood and urine for research.^18^ The study was approved by the Institutional Review Board at the National University of Singapore, and informed consent was obtained from all enrolled participants.

### Assessment of BP and covariates.

At recruitment, trained interviewers used structured questionnaires to conduct face-to-face interviews at the participants’ homes. Information on demographics, height, weight, weekly physical activity, smoking history, and alcohol consumption history was collected. Body mass index (BMI, kg/m^2^) was computed by dividing weight by height. Medical history, including physician-diagnosed hypertension, diabetes mellitus, coronary artery disease, and stroke, was also obtained.^18^ Information on age, height, weight, smoking, alcohol use, and medical history, including the use of any antihypertensive medication, was again updated during the follow-up I interview. Using standard protocols, the robustness of the self-reported, physician-diagnosed diabetes and hypertension had been validated in separate studies, and the accuracy was found to be 98.9% for diabetes and 88% for hypertension.^19,20^

BP measurements were taken for all participants who consented to home visits for blood/urine collection, as previously described.^21^ Briefly, trained staff used an automatic digital BP monitor (Omron HEM-705CP; Omron Corporation) to measure the participants’ BP while they were seated. Three measurements were taken at 3-minute intervals, and the average SBP and DBP were recorded after rounding up to the nearest integer. PP was computed by subtracting DBP from SBP.

### Diagnosis of CLTI.

Record linkage was performed with the Singapore MediClaim System, which is a nationwide database containing hospitalization data from all public and private hospitals in Singapore since 1990. Incident CLTI cases after the BP measurement visit were determined via surgical codes for LEA and surgical revascularization procedures listed in the Table of Surgical Procedures.^22^ Amputations performed for nonvascular causes, namely trauma, malignancy, peripheral neuropathy, necrotizing fasciitis, osteomyelitis, and osteonecrosis, were identified via the International Classification of Diseases Version 9 (ICD-9) and Version 10 (ICD-10) diagnosis codes and subsequently excluded from the present study, as previously described.^8^ Further record linkage was performed with the nationwide Registry of Births and Deaths for deceased participants. As of December 31, 2017, which was the censored date for both record linkages, only 41 participants in this cohort were known to be lost to follow-up due to reasons such as emigration, and as such, data capture could be considered as being virtually complete.

### Statistical analysis.

A total of 30,511 participants remained in the present analysis after excluding those who developed CLTI before the BP measurement, as well as those who had unrealistic BP measurements (SBP <80 or ≥250 mm Hg, DBP <40 or ≥150 mm Hg, or PP <10 mm Hg). Person-years were counted from the date of BP measurement to the date of CLTI development, loss to follow-up, death, or December 31, 2017, whichever came first.

Each BP parameter was categorized into ordinal groups of 10 mm Hg increment: SBP <120, 120–129, 130–139, 140–149, 150–159, ≥160 mm Hg; DBP <70, 70–79, 80–89, ≥90 mm Hg; and PP <40, 40–49, 50–59, 60–69, 70–79, ≥80 mm Hg. Baseline characteristics distributions were compared using extreme categories of BP parameters. The Fine-Gray method was applied to multivariable Cox proportional hazards models for a potential competing risk of death,^23^ allowing calculation of hazard ratios (HRs) and 95% confidence intervals (95% CIs) for each BP parameter in relation to CLTI risk. The lowest level of each parameter served as the reference group. Pearson’s pairwise correlations between SBP, DBP, and PP were calculated to assess the linear relationship among parameters.

The first model was adjusted for baseline confounders and vascular risk factors: age (years) at follow-up I, year of initial interview (1993–1995, 1996–1998), dialect (Hokkien, Cantonese), education (no formal education, primary school, secondary school or higher), weekly physical activity (<0.5 hours/wk, 0.5 to <4 hours/wk, ≥4 hours/wk), sex (male, female), BMI (kg/m^2^), smoking status (never, former, current), alcohol consumption status (never/monthly, weekly, daily), history of diabetes (no, yes), history of coronary artery disease (no, yes), history of stroke (no, yes), and use of antihypertensive medications (no, yes). In subsequent models, a second BP parameter (either SBP, DBP, or PP) was included as an ordinal variable, and the likelihood ratio test was used to examine whether the dual BP models were significantly different from single BP models. Sensitivity analyses were also performed by adding the second BP parameter as a continuous variable and also by excluding those who developed CLTI within 4 years of BP measurement.

A restricted spline analysis was used to examine the shape of the relationships between all three BP parameters and CLTI risk. The Akaike information criterion (AIC) was used to determine the final number of knots among three, four, or five knots that would fit the best approximating model, using the first knot (5%) as a reference. We further assessed how a basic predictive model including age, sex, history of diabetes (no, yes), and smoking status (never, former, current) is improved by adding each individual BP parameter (continuous). The concordance probability estimate (discriminatory power) was measured by Gönen and Heller’s K accounting for the presence of censoring.^24^ The AIC was used to determine the quality of statistical models balancing fit with complexity.

A stratified analysis was performed for vascular risk factors, namely history of diabetes (no, yes), age (<65, ≥65 years old), sex (male, female), BMI (<23, ≥23 kg/m^2^), smoking status (never, former, current), and use of antihypertensive medications (no, yes). Interaction was assessed by including the product term of PP and the interaction factor into the model.

Further classification of participants into four hypertension categories was performed as per the 2017 ACC/AHA guidelines for hypertension: (1) normal BP (SBP <120 mm Hg and DBP <80 mm Hg), (2) elevated BP (SBP = 120–129 mm Hg and DBP <80 mm Hg), (3) stage 1 hypertension (SBP = 130–139 mm Hg or DBP = 80–89 mm Hg), and (4) stage 2 hypertension (SBP ≥140 mm Hg or DBP ≥90 mm Hg).^17^ CLTI risk was evaluated in each category using normal BP as the reference group, and adjustment was further made for PP as an ordinal variable to determine whether the observed risk could be explained by the presence of a higher PP in stage 1 and stage 2 hypertension categories. Similarly, classification of participants was performed as per the 2023 European Society of Hypertension (ESH) guidelines for comparison.^25^

All statistical analyses were performed using SAS (version 3.81; SAS Institute, Inc). All presented *P* values are two-sided, and a *P* value <.05 was considered statistically significant.

## RESULTS

A total of 30,511 participants, accounting for 413,835 person-years, were included in this analysis. Characteristics of participants in the extreme categories of SBP, DBP, and PP are listed in Table I. Characteristics of participants in all categories are provided in Supplementary Table I (online only). For all BP parameters, participants with higher BP were more likely to be older, smoke, drink alcohol each week, be less educated, and have a history of diabetes, coronary artery disease, or stroke. Although more individuals engaged in ≥4 h/week of physical activity in higher SBP and DBP categories, this trend was less apparent in the PP categories (Supplementary Table I, online only). Pairwise comparisons of SBP, DBP, and PP via Pearson’s correlation coefficients revealed the highest correlation between SBP and PP (*r* = 0.88, *P* < .001), whereas DBP and PP had the lowest correlation (*r* = −0.30, *P* < .001).

After a mean follow-up period of 13.6 (±4.0) years, there were 407 incident CLTI cases. A restricted cubic spline analysis (Fig) revealed that SBP and PP had a linear relationship with CLTI risk (*P* for nonlinearity ≥ .55), whereas DBP had a somewhat J-shaped relationship with CLTI risk due to an increased risk at very low levels (*P* for nonlinearity = .0005). For SBP and PP, increasing pressures were associated with CLTI risk in a dose-dependent manner (Table II). Compared with the reference group, the multivariable-adjusted HRs (95% CIs) for the highest BP categories were 2.74 (1.86–4.04) for SBP ≥160 mm Hg and 5.24 (3.04–9.03) for PP ≥ 80 mm Hg; *P*s for trend for all were # .001. For DBP, compared with DBP <70 mm Hg, the risk decreased by about 10%, albeit nonsignificantly from 70 to 89 mm Hg, and increased thereafter, with HR (95% CI) being 1.74 (1.14–2.67) in the highest category for DBP >100 mm Hg. Compared with a base model using age, sex, smoking, and type 2 diabetes (AIC = 7625), adding PP improved the model fit (AIC = 7535) considerably more than SBP or DBP (AIC = 7560 and 7620, respectively). Compared with the concordance of the base model (K = 0.690), the discriminatory power was comparably improved by either PP (K = 0.716) or SBP (K = 0.715), whereas to a much lesser extent by DBP (K = 0.694).

Notably, in analyses that included two BP parameters, the dose-dependent association between PP and CLTI risk remained significant after adjusting for SBP or DBP (likelihood ratio tests comparing the PP-only model with PP + SBP or PP + DBP models, *P* ≥ .38). However, the association was no longer present in SBP and DBP after adjusting for PP (likelihood ratio tests comparing models without PP against models with PP, *P* < .0001), suggesting that PP was the strongest risk factor for CLTI risk. Results were similar whether the additional BP parameter was included as a continuous variable (Table II) or an ordinal variable (Supplementary Table II, online only). In the model that included both SBP and DBP, we found that the risk estimates for SBP after adjusting for DBP were higher than the case when SBP was the sole BP parameter (likelihood ratio test, *P* = .003) and that increased DBP, after adjusting for SBP, was actually protective against CLTI risk (*P* for trend < .0001), which also supported the associations observed for PP.

In stratified analyses for all other vascular risk factors (Supplementary Table III, online only), we found significant interactions between PP and history of diabetes (*P* for interaction = .02), whereby compared with those with diabetes, a higher PP conferred a greater risk of CLTI in patients without diabetes. There was no interaction between PP and sex, BMI, smoking status, or the use of antihypertensives (*P* for interaction ≥ .19). Sensitivity analyses using a 4-year lag time for CLTI outcomes also did not materially change our results (data not shown). Finally, an analysis was performed using the criteria for hypertension defined by the 2017 ACC/AHA guidelines (Table III). Compared with those with normal BP, as expected, those with stage 1 and stage 2 hypertension were found to have increased CLTI risk (HR for stage 1 hypertension, 1.74; 95% CI, 1.19–2.55; HR for stage 2 hypertension, 2.56; 95% CI, 1.73–3.77). However, after adjusting for PP, these associations were substantially attenuated with CIs extending below the null (HR for stage 1 hypertension, 1.07; 95% CI, 0.71–1.61; HR for stage 2 hypertension, 1.20; 95% CI, 0.77–1.88). A separate analysis was also performed using the 2023 ESH guidelines (Supplementary Table IV, online only), and again, associations were attenuated after adjusting for PP except in the grade 3 hypertension category (SBP ≥180 mm Hg and/or DBP ≥110 mm Hg).

## DISCUSSION

In this prospective population-based cohort of middle-aged and elderly Chinese adults in Singapore, we found that although SBP, DBP, and PP were individually associated with CLTI risk, after pairwise adjustment, the independent association with CLTI risk was strongest for PP. In addition, the association between hypertension stages 1 and 2 and CLTI risk in this cohort was substantially attenuated and no longer statistically significant after adjusting for PP.

In recent years, arterial stiffness has become increasingly recognized as a risk factor for atherosclerotic disease. The development of arterial stiffness is driven by many mechanisms, one of which is the age-related senescence of vascular endothelial cells and the loss of endothelial pro-genitor cells, which impairs calcium signaling, increases oxidative stress, and increases the expression of inflammatory cytokines.^26^ This stiffening of arteries, particularly large arteries, reduces the arteries’ ability to cushion changes in flow velocity and maintain a steady pressure, resulting in a greater pulse wave velocity and a higher pulsatile pressure.^27^ Exposure of small vessels to highly pulsatile pressure and flow could lead to microvascular damage in the foot and lower limb,^28^ thus resulting in CLTI that requires surgical intervention.

Calculated as the difference between SBP and DBP, PP is regarded as a surrogate for an estimation of BP variability in the artery and thus the distensibility of the artery.^29^ Indeed, the 2017 ACC/AHA hypertension guidelines acknowledge that although their guidelines still prioritize control of SBP and DBP, there is evidence to support the important role of PP.^17^ Our findings concurred with the few observational studies on the impact of arterial stiffness or PP on PAD. One cross-sectional study that previously examined associations between arterial stiffness and CLTI reported that compared with their age- and sex-matched controls, patients with CLTI had greater arterial stiffness as measured by brachial artery oscillometry.^30^ Other studies that examined arterial stiffness as measured by pulse wave velocity had similarly concurred that patients with PAD had greater arterial stiffness after adjusting for other vascular risk factors^31^ and that even in patients with well-controlled hypertension or normal BP, increased arterial stiffness was a significant predictor of PAD risk.^32^

To the best of our knowledge, ours is the first prospective cohort study that had specifically examined the association of PP, in combination with SBP and DBP, with the risk of CLTI. Several prospective studies that had examined associations between PP and less severe forms of PAD also concurred with our findings that PP increased the risk of disease.^14,15,33^ In the CALIBER UK cohort, which involved 1.25 million adults who did not have cardiovascular disease at baseline, the association with PP was strongest for PAD (HR per 10 mm Hg increase, 1.23; 95% CI, 1.20–1.27) than for other cardiovascular diseases included in that study.^33^ Another prospective cohort study involving 5885 Chinese participants also reported that a widened PP was significantly associated with PAD risk after adjusting for hypertension.^15^

In our study, although we found a similar predictive accuracy of CLTI for both PP and SBP, PP improved the model fit more substantially, and this concurred with the results from the National Health and Nutrition Examination Survey (NHANES, 1999–2002), which also included PP as the predictor related to BP in the final prediction model for PAD.^34^ Accordingly, although both BP parameters may be clinically useful for risk stratification, PP has greater relevance in relation to the underlying mechanisms because it reflects the causal pathway more accurately and explains a higher proportion of variance for CLTI. However, although PP might be a better choice for creating risk scores, SBP is an easier target to modify and might remain a practically more appropriate target for public health measures.

Interestingly, we observed a strong positive correlation between SBP and PP, but only a weak negative correlation between DBP and PP. Our finding was similar to the observation in the Framingham Heart Study cohort in 1999, which also noted that correlations among pairwise BP components were r = 0.87 for SBP-PP and r = 0.21 for PP-DBP.^35^ Although this may result from greater variability in SBP compared with DBP, it is also plausible that both PP and SBP increase in response to elevated large-artery stiffness and vascular resistance.

In addition, we also noted an interaction between PP and diabetes, whereby a raised PP conferred a greater risk on patients without diabetes than those with diabetes. An interaction between diabetes and hypertension had previously been reported in our study on diabetes and CLTI-associated lower limb amputation, whereby hypertension was only a risk factor in those without diabetes but not in those with diabetes.^8^ A similar interaction between PP and diabetes had also been previously reported by the Antihypertensive and Lipid-Lowering Treatment to Prevent Heart Attack Trial (ALLHAT) study, which noted that the association of PP with PAD-related events was significant for patients without diabetes mellitus but attenuated for patients with diabetes mellitus.^36^ Studies on the etiology of PAD have previously proposed a common pathway for diabetes and hypertension via the formation of advanced glycation end-products and increased uptake of low-density lipoproteins into macrophages.^37^ Hence, it is likely that raised PP and diabetes share common pathways in the development of CLTI.

The main strengths of this study include the prospective population-based design, the large sample size, the long follow-up period, the clinically significant end point of CLTI requiring surgical intervention rather than asymptomatic PAD, and the measurement of BP by trained interviewers at the participants’ homes, which would reduce the risk of white-coat hypertension due to the out-of-office setting and measurement errors due to improper BP measurement techniques.

However, some limitations should be acknowledged. First, given the observational study design, we could not rule out the possibility of residual confounding factors despite our attempt to adjust for established risk factors of PAD and demographic factors. Other residual confounders may include socioeconomic factors beyond education, chronic kidney disease, genetic pre-dispositions, and the use of statin and aspirin. However, to reduce such bias, we had confined our participants at recruitment to be Chinese adults residing in government purpose-built housing estates, which comprised 86% of the population at the time, and also included dialect as a covariate because dialect is a surrogate for the place of their ancestral origin in Southern China. Second, misclassification is possible because BP was measured during a single home visit, which would reduce but not eliminate white-coat hypertension, whereas hypertension should only be diagnosed by a consistently high BP measured on multiple occasions in the home setting. However, because BP was measured prospectively before the development of CLTI, any such misclassification should be nondifferential and likely to result in an underestimation of the risk estimates. Third, we did not have information on left ventricular ejection fraction or pulse wave velocity, and hence, it is possible that a widened PP in some participants reflected drastically reduced cardiac output due to severe heart failure rather than a measurement of their arterial stiffness. Fourth, in this study, CLTI was defined by the presence of severe PAD requiring surgical intervention and included cases with prior LEA or revascularization procedures. Hence, we were unable to include individuals who had received conservative or nonsurgical management, which may have led to an underestimation of CLTI incidence. We also recognize that such potential misclassification of cases as noncases within the cohort may have led us to underestimate the true risk estimates linking exposures to CLTI incidence. Finally, we did not collect data on the exact type of antihypertensive medication used, and as such, we are unable to examine whether certain types of medications may be protective against the development of arterial stiffness due to their mechanisms of action.

## CONCLUSIONS

In conclusion, in the present study, a widened PP was found to be a major risk factor for the development of CLTI. Clinically, our findings underscore the importance of maintaining normal PP in hypertension management and suggest that therapeutic strategies should balance systolic reduction with diastolic preservation to minimize the development of PAD. Therefore, our findings highlight the importance of further research to develop treatments that optimize systolic control while minimizing the risk of CLTI linked to excessively low diastolic pressures.

## Supplementary Material

1

## Figures and Tables

**Fig.**
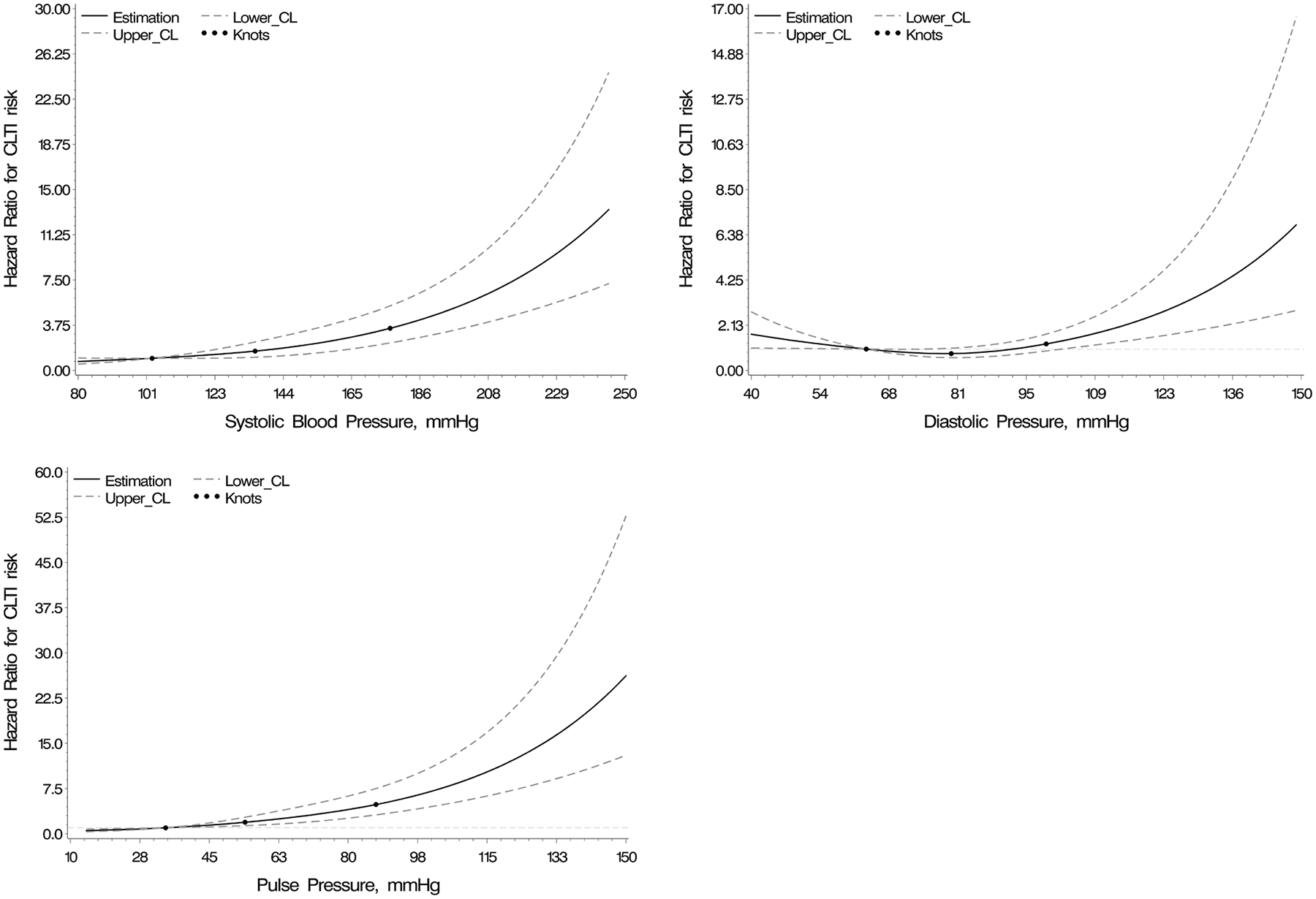
Restricted spline analyses for associations between BP parameters and CLTI risk. Models adjusted for age (year), sex (male, female), smoking status (never, former, current), and history of diabetes (no, yes). *BP*, Blood pressure; *CI*, confidence interval; *CLTI*, chronic limb-threatening ischemia; *HR*, hazard ratio.

**Table I.** Participant characteristics according to extreme categories of blood pressure (*BP*) in the Singapore Chinese Health Study

	SBP, mm Hg		DBP, mm Hg		PP, mm Hg	
	<120	≥160	<70	≥100	<40	≥80
No. of participants	7008	4605	4817	1520	4291	2844
No. of CLTI cases	38	119	61	35	17	102
BP, mm Hg, mean [SD]						
SBP	109.3 (7.7)	174.5 (13.6)	114.7 (16.2)	175.5 (19.8)	108.9 (10.6)	176.4 (17.1)
DBP	69.9 (6.9)	92.7 (11.1)	64.5 (4.1)	106.0 (6.5)	74.5 (9.4)	85.9 (12.6)
PP	39.4 (7.0)	81.8 (13.7)	50.2 (15.6)	69.5 (18.1)	34.5 (4.0)	90.5 (10.3)
Age, years, mean [SD]	59.8 (7.0)	66.9 (7.6)	63.4 (8.3)	62.9 (7.3)	58.1 (6.1)	69.5 (7.1)
BMI, kg/m^2^ mean [SD]	22.2 (3.4)	23.8 (3.6)	21.9 (3.4)	24.3 (3.7)	22.5 (3.6)	23.4 (3.6)
Sex, No. (%)						
Male	2419 (34.5%)	2255 (49.0%)	1494 (31.0%)	910 (60.0%)	1617 (37.7%)	1218 (42.8%)
Female	4589 (65.5%)	2350 (51.0%)	3323 (69.0%)	610 (40.0%)	2674 (62.3%)	1626 (57.2%)
Dialect group, No. (%)						
Hokkien	3630 (51.8%)	2258 (49.0%)	2564 (53.2%)	705 (46.4%)	2198 (51.2%)	1443 (50.7%)
Cantonese	3378 (48.2%)	2347 (51.0%)	2253 (46.8%)	815 (53.6%)	2093 (48.8%)	1401 (49.3%)
Level of education, No. (%)						
No formal education	1237 (17.6%)	1418 (30.8%)	1173 (24.3%)	347 (22.8%)	630 (14.7%)	1036 (36.4%)
Primary school	3026 (43.2%)	2141 (46.5%)	2162 (44.8%)	725 (47.7%)	1754 (40.8%)	1297 (45.6%)
Secondary or higher	2745 (39.2%)	1046 (22.7%)	1482 (30.8%)	448 (29.5%)	1907 (44.5%)	511 (18.0%)
Weekly physical activity, hours/wk, No. (%)						
<0.5	4561 (65.1%)	3071 (66.7%)	3215 (66.7%)	952 (62.6%)	2709 (63.2%)	1946 (68.4%)
0.5-<4	1615 (23.0%)	895 (19.4%)	1019 (21.2%)	353 (23.2%)	1064 (24.8%)	532 (18.7%)
≥4	832 (11.9%)	639 (13.9%)	583 (12.1%)	215 (14.1%)	518 (12.0%)	366 (12.9%)
Smoking, No. (%)						
Never	5210 (74.3%)	2858 (62.1%)	3441 (71.4%)	915 (60.2%)	3201 (74.6%)	1768 (62.2%)
Former	790 (11.3%)	915 (19.9%)	624 (13.0%)	299 (19.7%)	469 (10.9%)	564 (19.8%)
Current	1008 (14.4%)	832 (18.1%)	752 (15.6%)	306 (20.1%)	621 (14.5%)	512 (18.0%)
Alcohol drinking, No. (%)						
Never/monthly	6278 (89.6%)	4018 (87.3%)	4348 (90.2%)	1264 (83.2%)	3802 (88.6%)	2507 (88.1%)
Weekly	551 (7.9%)	377 (8.2%)	326 (6.8%)	180 (11.8%)	387 (9.0%)	210 (7.4%)
Daily	179 (2.5%)	210 (4.5%)	143 (3.0%)	76 (5.0%)	102 (2.4%)	127 (4.5%)
Medical history, No. (%)						
Diabetes	548 (7.8%)	1067 (23.2%)	684 (14.2%)	211 (13.9%)	275 (6.4%)	842 (30.0%)
Coronary artery disease	411 (5.9%)	473 (10.3%)	424 (8.8%)	109 (7.2%)	201 (4.7%)	365 (12.8%)
Stroke	138 (2.0%)	292 (6.3%)	161 (3.3%)	64 (4.1%)	70 (1.6%)	231 (8.1%)
Antihypertensive use	1159 (16.5%)	2478 (53.8%)	1134 (23.5%)	733 (48.2%)	694 (16.2%)	1681 (59.1%)

*BMI,* Body mass index; *CLTI,* chronic limb-threatening ischemia; *DBP*, diastolic blood pressure; *PP*, pulse pressure; *SBP*, systolic blood pressure.

**Table II.** Association between BP and CLTI risk in the Singapore Chinese Health Study

BP	Cases/No.	Rate per 100,000 person-year		HR (95% CI)^a^	
SBP, mm Hg			Model	Model + DBP^b^	Model + PP^b^
<120	38/7008	38.41	1.00	1.00	1.00
120–129	40/5352	53.00	1.05 (0.67–1.65)	1.18 (0.75–1.86)	0.80 (0.51–1.26)
130–139	67/5467	88.85	1.57 (1.05–2.35)	1.89 (1.23–2.88)	0.96 (0.62–1.47)
140–149	74/4767	114.55	1.76 (1.18–2.64)	2.27 (1.46–3.52)	0.87 (0.56–1.37)
150–159	69/3312	158.84	2.32 (1.54–3.50)	3.15 (1.98–4.99)	0.91 (0.56–1.49)
≥160	119/4605	212.57	2.74 (1.86–4.04)	4.16 (2.57–6.74)	0.64 (0.35–1.15)
*P* for trend			<.0001	<.0001	.33
DBP, mm Hg			Model	Model + SBP^b^	Model + PP^b^
<70	61/4817	92.21	1.00	1.00	1.00
70–79	126/10171	90.52	0.90 (0.66–1.22)	0.62 (0.45–0.85)	0.81 (0.60–1.11)
80–89	129/9885	95.27	0.90 (0.66–1.23)	0.43 (0.30–0.60)	0.71 (0.52–0.98)
90–99	56/4118	99.99	1.00 (0.69–1.46)	0.32 (0.20–0.50)	0.69 (0.47–1.01)
≥100	35/1520	176.52	1.74 (1.14–2.67)	0.34 (0.19–0.59)	1.02 (0.66–1.60)
*P* for trend			.01	<.0001	.09
PP, mm Hg			Model	Model + SBP^b^	Model + DBP^b^
<40	17/4291	27.36	1.00	1.00	1.00
40–49	50/7384	47.46	1.46 (0.84–2.53)	1.39 (0.79–2.44)	1.47 (0.84–2.55)
50–59	69/7363	67.63	1.71 (1.00–2.93)	1.56 (0.88–2.78)	1.73 (1.01–2.97)
60–69	88/5438	121.69	2.59 (1.52–4.42)	2.26 (1.23–4.18)	2.62 (1.53–4.50)
70–79	81/3191	201.58	3.64 (2.11–6.28)	3.04 (1.55–5.98)	3.70 (2.13–6.44)
≥80	102/2844	320.54	5.23 (3.04–9.03)	4.09 (1.89–8.88)	5.34 (3.05–9.34)
*P* for trend			<.0001	.002	<.0001

*BP*, Blood pressure; *CI*, confidence interval; *CLTI*, chronic limb-threatening ischemia; *DBP*, diastolic blood pressure; *HR*, hazard ratio; *PP*, pulse pressure; *SBP*, systolic blood pressure

a Model adjusted for age (year), year of interview (1993–1995, 1996–1998), dialect (Hokkien, Cantonese), education (no formal education, primary school, secondary school or higher), weekly physical activity (<0.5 hours/wk, 0.5 to <4 hours/wk, ≥4 hours/wk), sex (male, female), body mass index (<23, ≥23 kg/m^2^), smoking status (never, former, current), alcohol consumption status (never/monthly, weekly, daily), history of diabetes (no, yes), history of coronary artery disease (no, yes), history of stroke (no, yes), and usage of antihypertensives (no, yes).

b Further adjustment for SBP, DBP, and PP were as continuous variables.

**Table III.** Association between hypertension categories defined by 2017 ACC/AHA criteria and CLTI risk in the Singapore Chinese Health Study

Definitions	Blood pressure categories, mm Hg			
	Normal	Elevated	Stage 1	Stage 2
	SBP <120 and DBP <80	SBP 120–129 and DBP <80	SBP 130–139 or DBP 80–89	SBP ≥140 or DBP ≥90
Participants, No. (%)	6489 (21.3)	3526 (11.6)	12833 (42.0)	7663 (25.1)
PP, mean (SD)	39.9 (6.9)	51.4 (5.6)	57.9 (14.4)	70.3 (15.6)
Cases	33	31	176	167
HR (95% CI)^a^	1.00	1.21 (0.74–1.99)	1.74 (1.19–2.55)	2.56 (1.73–3.77)
PP-adjusted HR (95% CI)	1.00	0.98 (0.59–1.61)	1.07 (0.71–1.61)	1.20 (0.77–1.88)

a Model adjusted for age (year), year of interview (1993–1995, 1996–1998), dialect (Hokkien, Cantonese), (no formal education, primary school, secondary school or higher), weekly physical activity (<0.5 hours/wk, 0.5 to <4 hours/wk, ≥4 hours/wk), sex (male, female), body mass index (<23, ≥23 kg/m^2^), smoking status (never, former, current), alcohol consumption status (never/monthly, weekly, daily), history of diabetes (no, yes), history of coronary artery disease (no, yes), history of stroke (no, yes), and usage of antihypertensives (no, yes).

*ACC/AHA*, American College of Cardiology/American Heart Association; *CI*, confidence interval; *CLTI*, chronic limb-threatening ischemia; *DBP*, diastolic blood pressure; *HR*, hazard ratio; *PP*, pulse pressure; *SBP*, systolic blood pressure, *SD*, standard deviation.

## ARTICLE HIGHLIGHTS

- **Type of Research:** Prospective, single-center cohort study
- **Key Findings:** Among 30,636 participants aged 46–85 years, after adjustment for systolic or diastolic blood pressure, as well as other vascular risk factors, only pulse pressure remained independently associated with the risk of chronic limb-threatening ischemia (CLTI) in a stepwise manner.
- **Take Home Message:** This study found that only pulse pressure remained independently associated with the risk of CLTI after adjusting for other blood pressure parameters, suggesting the significance of reducing arterial stiffness to decrease CLTI risk.
