# Supplementary material for: Blood pressure parameters and the risk of chronic limb-threatening ischemia: The Singapore Chinese Health Study
Source: J Vasc Surg. Author manuscript; Available in PMC 2026 Jun 29. (PMC13312063; doi:10.1016/j.jvs.2025.12.088)
Supplement: 1 [file NIHMS2189223-supplement-1.pdf]

Supplementary Table I (online only). Participant characteristics according to categories of BP in the Singapore Chinese Health Study

	SBP (mm Hg)					
	<120	120 to <130	130 to <140	140 to <150	150 to <160	≥160
No. of participants	7008	5352	5467	4767	3312	4605
No. of CLTI cases	38	40	67	74	69	119
Blood pressure, mm Hg						
SBP	109.3 (7.7)	124.6 (2.9)	134.5 (2.9)	144.4 (2.8)	154.1 (2.8)	174.5 (13.6)
DBP	69.9 (6.9)	76.6 (6.7)	80.2 (7.6)	83.5 (8.1)	86.4 (8.7)	92.7 (11.1)
PP	39.4 (7.0)	48.0 (6.9)	54.3 (7.8)	60.9 (8.2)	67.6 (9.0)	81.8 (13.7)
Age, years	59.8 (7.0)	61.6 (7.3)	62.9 (7.5)	64.0 (7.5)	65.3 (7.6)	66.9 (7.6)
BMI, kg/m^2	22.2 (3.4)	23.1 (3.4)	23.4 (3.5)	23.5 (3.5)	23.7 (3.6)	23.8 (3.6)
Sex, No. (%)						
Male	2419 (34.5%)	2359 (44.1%)	2547 (46.6%)	2291 (48.1%)	1631 (49.3%)	2255 (49.0%)
Female	4589 (65.5%)	2993 (55.9%)	2920 (53.4%)	2476 (51.9%)	1685 (50.7%)	2350 (51.0%)
Dialect group, No. (%)						
Hokkien	3630 (51.8%)	2681 (50.0%)	2823 (51.6%)	2373 (49.8%)	1631 (49.3%)	2258 (49.0%)
Cantonese	3378 (48.2%)	2671 (50.0%)	2644 (48.4%)	2394 (50.2%)	1685 (50.7%)	2347 (51.0%)
Level of education, No. (%)						
No formal education	1237 (17.6%)	1048 (19.6%)	1143 (20.9%)	1131 (23.7%)	870 (26.3%)	1418 (30.8%)
Primary school	3026 (43.2%)	2390 (44.7%)	2509 (45.9%)	2176 (45.7%)	1547 (46.7%)	2141 (46.5%)
Secondary or higher	2745 (39.2%)	1914 (35.8%)	1815 (33.2%)	1460 (30.6%)	895 (27.0%)	1046 (22.7%)
Weekly physical activity, hours/wk, No. (%)						
<0.5	4561 (65.1%)	3421 (63.9%)	3450 (63.1%)	3097 (65.0%)	2154 (65.0%)	3071 (66.7%)
0.5-<4	1615 (23.0%)	1197 (22.4%)	1250 (22.9%)	1043 (21.9%)	671 (20.3%)	895 (19.4%)
≥4	832 (11.9%)	734 (13.7%)	767 (14.0%)	627 (13.1%)	487 (14.7%)	639 (13.9%)
Smoking, No. (%)						
Never	5210 (74.3%)	3741 (69.9%)	3735 (68.3%)	3112 (65.3%)	2097 (63.3%)	2858 (62.1%)
Former	790 (11.3%)	769 (14.4%)	908 (16.6%)	846 (17.7%)	662 (20.0%)	915 (19.9%)
Current	1008 (14.4%)	842 (15.7%)	824 (15.1%)	809 (17.0%)	553 (16.7%)	832 (18.1%)
Alcohol drinking, No. (%)						
Never/monthly	6278 (89.6%)	4689 (87.6%)	4822 (88.2%)	4187 (87.8%)	2912 (87.9%)	4018 (87.3%)
Weekly	551 (7.9%)	510 (9.5%)	497 (9.1%)	391 (8.2%)	268 (8.1%)	377 (8.2%)
Daily	179 (2.5%)	153 (2.9%)	148 (2.7%)	189 (4.0%)	132 (4.0%)	210 (4.5%)
Medical history, No. (%)						
Diabetes	548 (7.8%)	607 (11.3%)	769 (14.1%)	786 (16.5%)	618 (18.7%)	1067 (23.2%)
Hypertension	1471 (21.0%)	1920 (35.9%)	2557 (46.8%)	2601 (54.6%)	2044 (61.7%)	3294 (71.5%)
Coronary artery disease	411 (5.9%)	364 (6.8%)	428 (7.8%)	399 (8.4%)	283 (8.5%)	473 (10.3%)
Stroke	138 (2.0%)	156 (2.9%)	201 (3.7%)	222 (4.7%)	164 (5.0%)	292 (6.3%)
Antihypertensive use	1159 (16.5%)	1560 (29.2%)	2045 (37.4%)	2089 (43.8%)	1594 (48.1%)	2478 (53.8%)

	DBP (mm Hg)				
	<70	70 to <80	80 to <90	90 to <100	≥100
No. of participants	4817	10,171	9885	4118	1520
No. of CLTI cases	61	126	129	56	35
Blood pressure, mm Hg					
SBP	114.7 (16.2)	128.1 (15.7)	142.1 (16.2)	156.3 (16.9)	175.5 (19.8)

Supplementary Table I (online only). Continued.

	DBP (mm Hg)				
	<70	70 to <80	80 to <90	90 to <100	≥100
DBP	64.5 (4.1)	74.8 (2.8)	84.1 (2.8)	93.6 (2.8)	106.0 (6.5)
PP	50.2 (15.6)	53.3 (15.2)	58.0 (15.7)	62.8 (16.4)	69.5 (18.1)
Age, years	63.4 (8.3)	63.1 (7.9)	62.9 (7.5)	62.6 (7.5)	62.9 (7.3)
BMI, kg/m^2	21.9 (3.4)	23.0 (3.5)	23.5 (3.5)	24.0 (3.6)	24.3 (3.7)
Sex, No. (%)					
Male	1494 (31.0%)	4008 (39.4%)	4801 (48.6%)	2289 (55.6%)	910 (60.0%)
Female	3323 (69.0%)	6163 (60.6%)	5084 (51.4%)	1829 (44.4%)	610 (40.0%)
Dialect group, No. (%)					
Hokkien	2564 (53.2%)	5265 (51.8%)	4886 (49.4%)	1976 (48.0%)	705 (46.4%)
Cantonese	2253 (46.8%)	4906 (48.2%)	4999 (50.6%)	2142 (52.0%)	815 (53.6%)
Level of education, No. (%)					
No formal education	1173 (24.3%)	2313 (22.7%)	2149 (21.7%)	865 (21.0%)	347 (22.8%)
Primary school	2162 (44.8%)	4553 (44.8%)	4475 (45.3%)	1874 (45.5%)	725 (47.7%)
Secondary or higher	1482 (30.8%)	3305 (32.5%)	3261 (33.0%)	1379 (33.5%)	448 (29.5%)
Weekly physical activity, hours/wk, No. (%)					
<0.5	3215 (66.7%)	6651 (65.4%)	6331 (64.1%)	2605 (63.2%)	952 (62.6%)
0.5-<4	1019 (21.2%)	2176 (21.4%)	2218 (22.4%)	905 (22.0%)	353 (23.2%)
≥4	583 (12.1%)	1344 (13.2%)	1336 (13.5%)	608 (14.8%)	215 (14.1%)
Smoking, No. (%)					
Never	3441 (71.4%)	7128 (70.1%)	6603 (66.8%)	2666 (64.7%)	915 (60.2%)
Former	624 (13.0%)	1512 (14.9%)	1695 (17.2%)	760 (18.5%)	299 (19.7%)
Current	752 (15.6%)	1531 (15.0%)	1587 (16.0%)	692 (16.8%)	306 (20.1%)
Alcohol drinking, No. (%)					
Never/monthly	4348 (90.2%)	9097 (89.4%)	8639 (87.4%)	3558 (86.4%)	1264 (83.2%)
Weekly	326 (6.8%)	798 (7.9%)	898 (9.1%)	192 (9.5%)	180 (11.8%)
Daily	143 (3.0%)	276 (2.7%)	348 (3.5%)	168 (4.1%)	76 (5.0%)
Medical history, No. (%)					
Diabetes	684 (14.2%)	1464 (14.4%)	1473 (14.9%)	562 (13.7%)	211 (13.9%)
Hypertension	1382 (28.7%)	3911 (38.5%)	4983 (50.4%)	2504 (60.8%)	1107 (72.8%)
Coronary artery disease	424 (8.8%)	806 (7.9%)	730 (7.4%)	289 (7.02%)	109 (7.2%)
Stroke	161 (3.3%)	393 (3.9%)	386 (3.9%)	171 (4.2%)	64 (4.1%)
Antihypertensive use	1134 (23.5%)	3244 (31.9%)	3964 (40.1%)	1850 (44.9%)	733 (48.2%)

	PP (mm Hg)					
	<40	40 to <50	50 to <60	60 to <70	70 to <80	≥80
No. of participants	4291	7384	7363	5438	3191	2844
No. of CLTI cases	17	50	69	88	81	102
Blood pressure, mm Hg						
SBP	108.9 (10.6)	122.6 (10.3)	135.2 (10.9)	146.4 (11.3)	158.1 (12.2)	176.4 (17.1)
DBP	74.5 (9.4)	77.9 (9.6)	80.8 (10.3)	82.2 (10.8)	84.0 (11.8)	85.9 (12.6)
PP	34.5 (4.0)	44.7 (2.8)	54.4 (2.9)	64.2 (2.9)	74.1 (2.9)	90.5 (10.3)
Age, years	58.1 (6.1)	60.1 (6.6)	62.7 (7.3)	65.2 (7.3)	67.2 (7.2)	69.5 (7.1)
BMI, kg/m^2	22.5 (3.6)	23.0 (3.4)	23.3 (3.6)	23.5 (3.6)	23.6 (3.6)	23.4 (3.6)
Sex, No. (%)						
Male	1617 (37.7%)	3323 (45.0%)	3435 (46.7%)	2491 (45.8%)	1418 (44.4%)	1218 (42.8%)
Female	2674 (62.3%)	4061 (55.0%)	3928 (53.3%)	2947 (54.2%)	1773 (55.6%)	1626 (57.2%)
Dialect group, No. (%)						

(Continued on next page)

Supplementary Table I (online only). Continued.

	PP (mm Hg)					
	<40	40 to <50	50 to <60	60 to <70	70 to <80	≥80
Hokkien	2198 (51.2%)	3721 (50.4%)	3685 (50.0%)	2754 (50.6%)	1595 (50.0%)	1443 (50.7%)
Cantonese	2093 (48.8%)	3663 (49.6%)	3678 (50.0%)	2684 (49.4%)	1596 (50.0%)	1401 (49.3%)
Level of education, No. (%)						
No formal education	630 (14.7%)	1259 (17.1%)	1539 (20.9%)	1404 (25.8%)	979 (30.7%)	1036 (36.4%)
Primary school	1754 (40.8%)	3277 (44.4%)	3399 (46.2%)	2591 (47.7%)	1471 (46.1%)	1297 (45.6%)
Secondary or higher	1907 (44.5%)	2848 (38.6%)	2425 (32.9%)	1443 (26.5%)	741 (23.2%)	511 (18.0%)
Weekly physical activity, hours/wk, No. (%)						
<0.5	2709 (63.2%)	4643 (62.9%)	4708 (63.9%)	3605 (66.3%)	2143 (67.2%)	1946 (68.4%)
0.5-<4	1064 (24.8%)	1750 (23.7%)	1659 (22.5%)	1071 (19.7%)	585 (18.6%)	532 (18.7%)
≥4	518 (12.0%)	991 (13.4%)	996 (13.5%)	762 (14.0%)	453 (14.2%)	366 (12.9%)
Smoking, No. (%)						
Never	3201 (74.6%)	5280 (71.5%)	4971 (67.5%)	3523 (64.8%)	2010 (63.0%)	1768 (62.2%)
Former	469 (10.9%)	1029 (13.9%)	1170 (15.9%)	1003 (18.4%)	655 (20.5%)	564 (19.8%)
Current	621 (14.5%)	1075 (14.6%)	1222 (16.6%)	912 (16.8%)	526 (16.5%)	512 (18.0%)
Alcohol drinking, No. (%)						
Never/monthly	3802 (88.6%)	6455 (87.4%)	6470 (87.9%)	4816 (88.6%)	2856 (89.5%)	2507 (88.1%)
Weekly	387 (9.0%)	726 (9.8%)	643 (8.7%)	414 (7.6%)	214 (6.7%)	210 (7.4%)
Daily	102 (2.4%)	203 (2.8%)	250 (3.4%)	208 (3.8%)	121 (3.8%)	127 (4.5%)
Medical history, No. (%)						
Diabetes	275 (6.4%)	644 (8.7%)	921 (12.5%)	977 (18.0%)	736 (23.1%)	842 (30.0%)
Hypertension	916 (21.4%)	2482 (33.6%)	3324 (45.1%)	3051 (56.1%)	2023 (63.4%)	2091 (73.5%)
Coronary artery disease	201 (4.7%)	428 (5.8%)	570 (7.7%)	471 (8.7%)	323 (10.1%)	365 (12.8%)
Stroke	70 (1.6%)	182 (2.5%)	243 (3.3%)	258 (4.7%)	189 (5.9%)	231 (8.1%)
Antihypertensive use	694 (16.2%)	1969 (26.7%)	2576 (35.0%)	2434 (44.8%)	1571 (49.2%)	1681 (59.1%)

BMI, Body mass index; *BP,* blood pressure; *CLTI,* chronic limb-threatening ischemia; *DBP,* diastolic blood pressure; *PP,* pulse pressure; *SBP,* systolic blood pressure.

Supplementary Table II (online only). Association between BP and CLTI risk in the Singapore Chinese Health Study

BP	HR (95% CI)[a]				
SBP, mm Hg	Model	Model + DBP (ordinal variable)[b]	Model + DBP[b] (categorical variable)	Model + PP (ordinal variable)[b]	Model + PP[b] (categorical variable)
<120	1.00	1.00	1.00	1.00	1.00
120-129	1.05 (0.67-1.65)	1.18 (0.75-1.86)	1.30 (0.82-2.05)	0.76 (0.48-1.21)	0.84 (0.51-1.38)
130-139	1.57 (1.05-2.35)	1.90 (1.24-2.89)	2.19 (1.42-3.36)	0.88 (0.56-1.38)	1.03 (0.61-1.71)
140-149	1.76 (1.18-2.64)	2.30 (1.49-3.55)	2.75 (1.76-4.29)	0.77 (0.47-1.26)	0.90 (0.52-1.57)
150-159	2.32 (1.54-3.50)	3.21 (2.03-5.05)	3.83 (2.40-6.12)	0.77 (0.45-1.35)	0.88 (0.49-1.61)
≥160	2.74 (1.86-4.04)	4.25 (2.66-6.81)	4.78 (2.96-7.72)	0.63 (0.34-1.18)	0.68 (0.36-1.29)
P for trend	<.0001	<.0001	<.0001	.62	.51
DBP, mm Hg	Model	Model + SBP	Model + SBP	Model + PP	Model + PP
<70	1.00	1.00	1.00	1.00	1.00
70-79	0.90 (0.66-1.22)	0.65 (0.47-0.90)	0.64 (0.46-0.89)	0.82 (0.60-1.11)	0.82 (0.60-1.11)
80-89	0.90 (0.66-1.23)	0.46 (0.32-0.66)	0.45 (0.31-0.64)	0.72 (0.52-0.98)	0.72 (0.52-0.99)
90-99	1.00 (0.69-1.46)	0.38 (0.24-0.60)	0.38 (0.24-0.59)	0.70 (0.48-1.02)	0.70 (0.48-1.02)
≥100	1.74 (1.14-2.67)	0.55 (0.33-0.92)	0.56 (0.33-0.94)	1.08 (0.70-1.68)	1.08 (0.70-1.69)
P for trend	.01	<.0001	<.0001	.07	.07
PP, mm Hg	Model	Model + SBP	Model + SBP	Model + DBP	Model + DBP
<40	1.00	1.00	1.00	1.00	1.00
40-49	1.46 (0.84-2.53)	1.52 (0.84-2.73)	1.52 (0.84-2.73)	1.52 (0.87-2.64)	1.52 (0.87-2.64)
50-59	1.71 (1.00-2.93)	1.80 (0.94-3.46)	1.80 (0.94-3.46)	1.81 (1.05-3.11)	1.81 (1.05-3.11)
60-69	2.59 (1.52-4.42)	2.84 (1.41-5.70)	2.84 (1.41-5.70)	2.74 (1.60-4.70)	2.74 (1.60-4.70)
70-79	3.64 (2.11-6.28)	4.42 (2.10-9.33)	4.42 (2.10-9.33)	3.82 (2.20-6.66)	3.82 (2.20-6.66)
≥80	5.23 (3.04-9.03)	7.28 (3.30-16.11)	7.28 (3.30-16.11)	5.58 (3.19-9.75)	5.58 (3.19-9.75)
P for trend	<.0001	<.0001	<.0001	<.0001	<.0001

BP, Blood pressure; *CI*, confidence interval; *CLTI*, chronic limb-threatening ischemia; *DBP*, diastolic blood pressure; *HR*, hazard ratio; *PP*, pulse pressure; *SBP*, systolic blood pressure.
[a]Model adjusted for age (year), year of interview (1993-1995, 1996-1998), dialect (Hokkien, Cantonese), (no formal education, primary school, secondary school or higher), weekly physical activity (<0.5 hours/wk, 0.5 to <4 hours/wk, ≥4 hours/wk), sex (male, female), body mass index (<23, ≥23 kg/m^2), smoking status (never, former, current), alcohol consumption status (never/monthly, weekly, daily), history of diabetes (no, yes), history of coronary artery disease (no, yes), history of stroke (no, yes), and usage of antihypertensives (no, yes).
[b]Further adjustments for SBP, DBP, and PP were as ordinal or categorical variables. SBP reference group: 120-129. DBP reference group: 70-79. PP reference group: 40-49.

Supplementary Table III (online only). Association between PP and CLTI risk in the Singapore Chinese Health Study, stratified by vascular risk factors

	Cases	HR (95% CI)[a]	Cases	HR (95% CI)[a]	*P* for interaction
By diabetes	No diabetes		Had diabetes		.02
<40	7	1.00	10	1.00	
40-49	24	1.93 (0.83-4.50)	26	1.05 (0.51-2.19)	
50-59	35	2.83 (1.25-6.44)	34	0.97 (0.48-1.98)	
60-69	29	3.30 (1.42-7.69)	59	1.76 (0.89-3.48)	
70-79	23	4.88 (2.03-11.73)	58	2.38 (1.20-4.75)	
≥80	35	10.00 (4.24-23.61)	67	2.97 (1.49-5.94)	
By sex	Male		Female		.26
<40	9	1.00	8	1.00	
40-49	32	1.53 (0.73-3.22)	18	1.27 (0.55-2.92)	
50-59	41	1.62 (0.78-3.35)	28	1.77 (0.80-3.93)	
60-69	51	2.56 (1.24-5.28)	37	2.52 (1.14-5.55)	
70-79	36	2.78 (1.30-5.93)	45	4.57 (2.07-10.05)	
≥80	44	4.52 (2.13-9.60)	58	5.88 (2.66-13.01)	
By BMI	<23 kg/m^2		≥23 kg/m^2		.32
<40	6	1.00	11	1.00	
40-49	22	2.04 (0.82-5.04)	28	1.08 (0.54-2.18)	
50-59	35	2.89 (1.20-6.96)	34	1.08 (0.55-2.15)	
60-69	39	3.79 (1.56-9.20)	49	1.83 (0.94-3.58)	
70-79	26	4.00 (1.59-10.09)	55	3.06 (1.56-5.99)	
≥80	48	8.26 (3.36-20.32)	54	3.53 (1.78-6.98)	
By smoking status	Non-/former smoker		Current smoker		.35
<40	12	1.00	5	1.00	
40-49	32	1.35 (0.69-2.63)	18	1.59 (0.58-4.32)	
50-59	57	2.10 (1.12-3.93)	12	0.91 (0.32-2.63)	
60-69	65	2.81 (1.49-5.29)	23	2.15 (0.79-5.87)	
70-79	68	4.36 (2.30-8.25)	13	2.11 (0.71-6.25)	
≥80	82	5.89 (3.10-11.17)	20	4.03 (1.42-11.45)	
By antihypertensives	No antihypertensives		On sntihypertensives		.19
<40	11	1.00	6	1.00	
40-49	34	1.68 (0.85-3.30)	16	0.92 (0.36-2.35)	
50-59	34	1.59 (0.80-3.16)	35	1.47 (0.62-3.51)	
60-69	45	2.81 (1.42-5.56)	43	1.83 (0.77-4.32)	
70-79	32	3.46 (1.69-7.10)	49	2.96 (1.25-7.01)	
≥80	35	5.91 (2.89-12.13)	67	3.83 (1.63-9.01)	

BMI, body mass index; *CI,* confidence interval; *CLTI,* chronic limb-threatening ischemia; *HR,* hazard ratio; *PP,* pulse pressure.
P for trend ≤.002 for all.
P for correlation ≤.0002 for all.
[a]Model adjusted for age (year), year of interview (1993-1995, 1996-1998), dialect (Hokkien, Cantonese), (no formal education, primary school, secondary school or higher), weekly physical activity (<0.5 hours/wk, 0.5 to <4 hours/wk, ≥4 hours/wk), plus sex (male, female), body mass index (<23, ≥23 kg/m^2), smoking status (never, former, current), alcohol consumption status (never/monthly, weekly, daily), history of diabetes (no, yes), history of coronary artery disease (no, yes), history of stroke (no, yes), and usage of antihypertensives (no, yes), except the stratified variable.

Supplementary Table IV (online only). Association between hypertension categories defined by 2023 ESC/ESH criteria and CLTI risk in the Singapore Chinese Health Study

	Definition	Participants	PP, mean (SD)	Cases	HR (95% CI)[a]	PP-adjusted, HR (95% CI)[a]
Normal BP						
Optimal	SBP <120 and DBP <80	6489	39.9 (6.9)	33	1.00	1.00
Normal	SBP 120-129 and DBP 80-84	9636	53.7 (13.1)	106	1.49 (1.00-2.21)	1.05 (0.70-1.60)
High-normal	SBP 130-139 and/or DBP 85-89	6827	60.0 (12.8)	101	1.82 (1.22-2.73)	1.17 (0.76-1.80)
Hypertension						
Grade 1	SBP 140-159 and/or DBP 90-99	5894	67.9 (13.4)	113	2.24 (1.50-3.34)	1.22 (0.78-1.91)
Grade 2	SBP 160-179 and/or DBP 100-109	1393	79.3 (15.5)	35	2.99 (1.82-4.88)	1.25 (0.71-2.21)
Grade 3	SBP ≥180 and/or DBP ≥110	272	89.4 (18.8)	19	9.00 (5.06-16.01)	2.92 (1.47-5.79)
Isolated systolic	SBP ≥140 and DBP <90	7684	72.0 (12.5)	174	2.25 (1.49-3.39)	0.94 (0.56-1.60)
Isolated diastolic	SBP <140 and DBP ≥90	638	41.1 (5.4)	3	0.82 (0.25-2.71)	0.80 (0.24-2.62)

CI, Confidence interval; *CLTI,* chronic limb-threatening ischemia; *DBP,* diastolic blood pressure, *ESC,* European Society of Cardiology; *ESH,* European Society of Hypertension; *HR,* hazard ratio; *PP,* pulse pressure; *SBP,* systolic blood pressure; *SD,* standard deviation.
[a]Model adjusted for age (year), year of interview (1993-1995, 1996-1998), dialect (Hokkien, Cantonese), (no formal education, primary school, secondary school or higher), weekly physical activity (<0.5 hours/wk, 0.5 to <4 hours/wk, ≥4 hours/wk), sex (male, female), body mass index (<23, ≥23 kg/m^2), smoking status (never, former, current), alcohol consumption status (never/monthly, weekly, daily), history of diabetes (no, yes), history of coronary artery disease and stroke (no, yes), and usage of antihypertensives (no, yes).
